# Management of periampullary variceal bleeding with a biliary stent and endoscopic ultrasound-guided cyanoacrylate injection

**DOI:** 10.1055/a-2839-9457

**Published:** 2026-04-15

**Authors:** Foke van Delft, Eric T. T. L. Tjwa, Kirsten Boonstra

**Affiliations:** 1 6034 Department of Gastroenterology and Hepatology, Radboud University Medical Center, Nijmegen, The Netherlands

Portal cavernoma cholangiopathy is caused by prolonged portal hypertension with compression and distortion of bile ducts by periportal collateral veins. Some patients with ischemic bile duct injury or gallstones require an endoscopic biliary intervention in which the risk of peri-procedural bleeding can be significant 1 2 . Endoscopic ultrasound guided vascular interventions can be helpful to manage bleeding complications 3 .

A 56-year old man with non-cirrhotic portal hypertension caused by splanchnic vein thrombosis presented with elevated liver enzymes in a cholestatic pattern. Cross-sectional imaging showed dilated periportal varices compressing the distal common bile duct. Because of colicky pain, magnetic resonance cholangiopancreatography was undertaken, which demonstrated a 5 mm gallstone in the common bile duct. A large periampullary varix was detected by the radiologist of our multidisciplinary team, who alerted us, expecting us to perform an endoscopic bile duct intervention.

During endoscopic retrograde cholangiography, the major papilla was carefully dilated with an 8 mm dilatation balloon to avoid electrocautery induced thermal injury. The same balloon was used to extract the gallstone with the idea to minimize radial stress on the bile duct when using a regular stone extraction balloon Stone extraction, however, precipitated a significant bleeding ( Fig. 1 ) from the distal bile duct, which we could easily control with a fully covered biliary metal stent ( Video 1 ). Two weeks later we performed endoscopic ultrasonography to assess the relationship between the biliary stent and the varix. With a 22 G FNA needle, we injected 1.5 ml of cyanoacrylate/lipiodol (1:1) into the culprit varix until the Doppler signal disappeared ( Video 1 ). Fluoroscopy showed a cyanoacrylate cast in the varix after which the biliary stent safely could be removed. Occlusion cholangiography showed no other intra-ductal abnormalities. A balloon sweep did not provoke any further bleeding. The patient was discharged the next day in good condition and remained stable during follow-up.

**Fig. 1:**
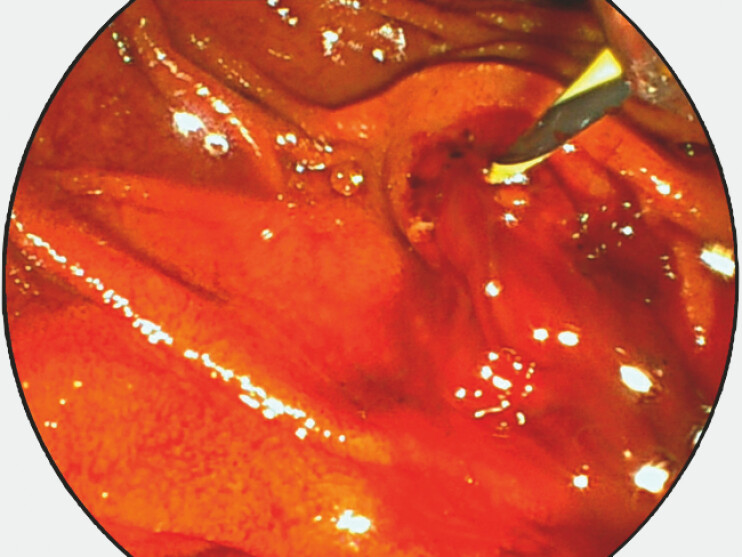
Variceal bleeding in the common bile duct after gallstone extraction.

Bleeding in the common bile duct. Placement of a fully covered metal stent. Endoscopic ultrasound-guided cyanoacrylate injection in the adjoining periampullary varix. Safe removal of the metal stent.Video 1

Endoscopy_UCTN_Code_TTT_1AR_2AC

Endoscopy_UCTN_Code_TTT_1AS_2AL

## References

[1] Pinto E Elkrief L Hernandez-Gea V Portal cavernoma cholangiopathy: A systematic review of current understanding, clinical significance, and management Hepatol Commun 2025 9 e0833 10.1097/HC9.0000000000000833 41085547 PMC12520217

[2] Franceschet I Zanetto A Ferrarese A Therapeutic approaches for portal biliopathy: A systematic review World J Gastroenterol 2016 22 9909 9920 10.3748/wjg.v22.i45.9909 28018098 PMC5143758

[3] Farkas ZC Chugh P Frager S Periampullary variceal bleeding: An atypical complication of portal hypertension Case Rep Gastrointest Med 2018 2018 4.643695E6 10.1155/2018/4643695 PMC5960554 29854492

